# Pyruvate carboxylase deficiency type C; variable presentation and beneficial effect of triheptanoin

**DOI:** 10.1002/jmd2.12405

**Published:** 2023-12-28

**Authors:** I. Bernhardt, L. Van Dorp, M. Dixon, M. McSweeney, C. Gan, J. Baruteau, A. Chakrapani

**Affiliations:** ^1^ Department of Paediatric Metabolic Medicine Great Ormond Street Hospital for Children London UK; ^2^ Dietetics Department Great Ormond Street Hospital for Children London UK; ^3^ Great Ormond Street Institute of Child Health, University College London London UK

## Abstract

Pyruvate carboxylase is a mitochondrial enzyme essential for the tricarboxylic acid cycle (TCA), gluconeogenesis and fatty‐acid synthesis. Pyruvate carboxylase deficiency (PCD) mostly presents with life‐limiting encephalopathy (types A/B). A milder type C presentation is rare, with a comparatively favourable prognosis. Therapies remain essentially supportive. Triheptanoin is an odd‐chain triglyceride, with the potential to replenish TCA intermediates (anaplerosis), and its metabolites cross the blood–brain‐barrier. Outcomes of triheptanoin treatment in PCD types A/B have been disappointing, but have not been reported in type C. Here, we present two new patients with PCD type C, and report the response to treatment with triheptanoin in one. Patient 1 (P1) presented with neonatal‐onset lactic acidosis and recurrent symptomatic lactic acidosis following exercise and during illnesses, with frequent hospitalisations. Speech development was delayed. MRI‐brain showed delayed cerebral myelination. Patient 2 (P2) presented with episodic ketoacidosis, hyperlactataemia and hypoglycaemia at 2 years of age, with gross motor delay and mild global volume loss on MRI brain. Treatment with triheptanoin was commenced in P1 at 3 years of age with up‐titration to 35 mL/day (25% of daily energy intake) over 6 months, due to transient diarrhoea. Dietary long‐chain triglycerides were restricted, with fat‐soluble vitamin supplementation. Subsequently, hospitalisations during intercurrent illnesses decreased, post‐exertional hyperlactataemia resolved and exercise tolerance improved. Continued developmental progress was observed, and repeat MRI 18 months after initiation showed improved myelination. Triheptanoin was well‐tolerated and appeared efficacious during 2 years' follow‐up, and has potential to restore energy homeostasis and myelin synthesis in PCD type C.

**Synopsis**

Triheptanoin treatment in a patient with pyruvate carboxylase deficiency (PCD) type C was associated with reduced hospitalisations, resolution of post‐exertional hyperlactataemia and improved myelination and development during 2 years' follow‐up.

## 1 INTRODUCTION

Pyruvate carboxylase (PC, EC#6.4.1.1) is a biotin‐dependent enzyme encoded by the *PC* gene, which catalyses the conversion of pyruvate to oxaloacetate within the mitochondria.^1^ This reaction has three key metabolic roles; replenishing tricarboxylic acid (TCA) cycle intermediates (termed anaplerosis), gluconeogenesis, and acetyl‐CoA export from mitochondria, for fatty‐acid synthesis. Pyruvate carboxylase deficiency (PCD; OMIM#266150) due to bi‐allelic *PC* variants, is a rare disorder (1:250 000 live‐births), associated with lactic acidosis, hypoglycaemia and hypomyelination due to impairment of the TCA cycle, gluconeogenesis, and fatty acid synthesis, respectively.^1^, ^2^

Three phenotypes of PCD are recognised; types A and B present in infancy with lactic acidosis and severe neurological manifestations, and have a poor prognosis.^1^, ^2^ A rare attenuated form (type C) was first described in 1991, and only 11 patients have been reported (seven molecularly confirmed).^3^, ^4^, ^5^, ^6^, ^7^, ^8^, ^9^, ^10^, ^11^ It is characterised by episodic hyperlactataemia and ketoacidosis, and has a favourable outcome, with relatively mild developmental delay and variable myelination abnormalities.

Treatment of PCD is generally supportive, with a lack of effective disease‐modifying therapies. Biotin is frequently trialled, with minimal clinical effect in patients with PCD types A/B, and few reports of benefit in PCD type C.^4^, ^12^ Supplementation with TCA intermediates citrate and aspartate has been shown to stabilise biochemical derangements; however, they do not appear to correct biochemical abnormalities in cerebrospinal fluid (CSF) or alter the neurological outcome.^13^

Triheptanoin is a synthetic odd‐carbon medium‐chain triglyceride comprising a glycerol backbone with three seven‐carbon (C7) fatty acids, and hydrolysis yields C5‐fatty acids or propionyl‐CoA (C3) in addition to acetyl‐CoA (C2).^14^ Propionyl‐CoA is metabolised to succinyl‐CoA which may then replenish the TCA cycle. Triheptanoin has been widely used in the treatment of long‐chain fatty acid oxidation disorders (LC‐FAOD), with evidence for improved metabolic stability and exercise tolerance.^15^, ^16^, ^17^

Clinical outcomes of triheptanoin treatment for PCD type B have been poor.^14^, ^18^ However biochemical improvements and improved myelination were noted in one individual.^14^ Encouragingly, C5‐ketones were demonstrated in CSF, suggesting that triheptanoin metabolites cross the blood–brain‐barrier.^14^ Therefore, triheptanoin has potential to restore cerebral energy homeostasis and improve myelination in PCD. Improvements in speech and eye contact have been reported in one patient with PCD type C treated with triheptanoin^10^; however, the outcome of treatment is yet to be described in detail. Here we present two new patients with PCD type C, and describe the beneficial effect of triheptanoin treatment in one.

## 2 CASE REPORTS

### 2.1 Patient 1

Patient 1 (P1) was the second child to non‐consanguineous parents. He was born at 38/40 following induction of labour. Grunting respirations were noted at 18 h of age, and he was found to have severe lactic acidosis and hypoglycaemia (Table 1), without hyperammonaemia. Reflexes and tone were normal, and there were no dysmorphic features or hepatomegaly. Hypoglycaemia and acidosis resolved following intravenous glucose and sodium bicarbonate infusions. Biotin (10 mg/day) and thiamine (50 mg/day) were commenced, and enteral feeding was established prior to discharge home on day five.

**TABLE 1:** Summary of clinical features and genotype in two patients with pyruvate carboxylase deficiency type C.

	Patient 1	Patient 2
*PC* genotype	c.1358G>A, p.(Arg453Gln); c.865_870del, p.(Thr289_Arg290del)	c.2767C>G, p.(Gln923Glu); c.2800del, p.(Gln934Argfs*35)
Age at presentation	18 h	2 years
Clinical presentation	Grunting respiration, hypoglycaemia and metabolic acidosis	Vomiting, laboured breathing, shock, reduced alertness and metabolic acidosis
Venous blood gas results at presentation (reference range)	pH 7.048 (7.35–7.45)	pH 6.88 (7.35–7.45)
	Bicarbonate 10.4 mmol/L (22–28)	Bicarbonate 5.1 mmol/L (22–28)
	Base excess −24.9 (−3 − +3)	Base excess −26.7 (−3 − +3)
	Lactate 18 mmol/L (<2.2 mmol/L)	Lactate 6.4 mmol/L (<2.2 mmol/L)
Hypoglycaemia at the time of initial metabolic crisis	Yes (1.1 mmol/L)	No (hypoglycaemia present during second acute crisis)
Hyperammonaemia	No	No
Urine organic acids	Increased excretion of lactate and 3‐hydroxybutyrate, milder elevations of pyruvate and acetoacetate.	Strongly increased ketones, lactate and pyruvate, moderately increased 2‐oxoisocaproate and 2‐hydroxybutyrate.
	Intermittently increased excretion of 2‐oxoglutarate, fumarate, 2‐hydroxybutyrate and 2‐oxoisocaproate	
Plasma amino acids	Alanine 555 μmol/L (150–450)	Alanine 532 μmol/L (150–450)
Median values (reference range)	Glutamine 419 μmol/L (480–800)	Glutamine 465 μmol/L (480–800)
	Lysine 176 μmol/L (100–300)	Lysine 132 μmol/L (100–300)
	Citrulline 23 μmol/L (8–57)	Citrulline N/A
	Aspartate 3 μmol/L (20–42)	Aspartate 2 μmol/L (20–42)
Total episodes of significant metabolic acidosis*	6	2
Exertional dyspnoea (peak lactate post‐exertion)	Yes (6.2 mmol/L)	No (2.6 mmol/L)
Development	Delayed speech, broad‐based gait with toe‐walking	Gross motor delay, unsteady gait with frequent falls
MRI brain (at 3 years of age)	Globally delayed cerebral myelination.	Mild global volume loss, left frontotemporal cystic lesion
Age at last follow‐up	5.5 years	3.6 years

Abbreviation: N/A, not assessed.

* Significant acidosis defined as requiring hospital admission and intravenous therapy.

**FIGURE 1:**
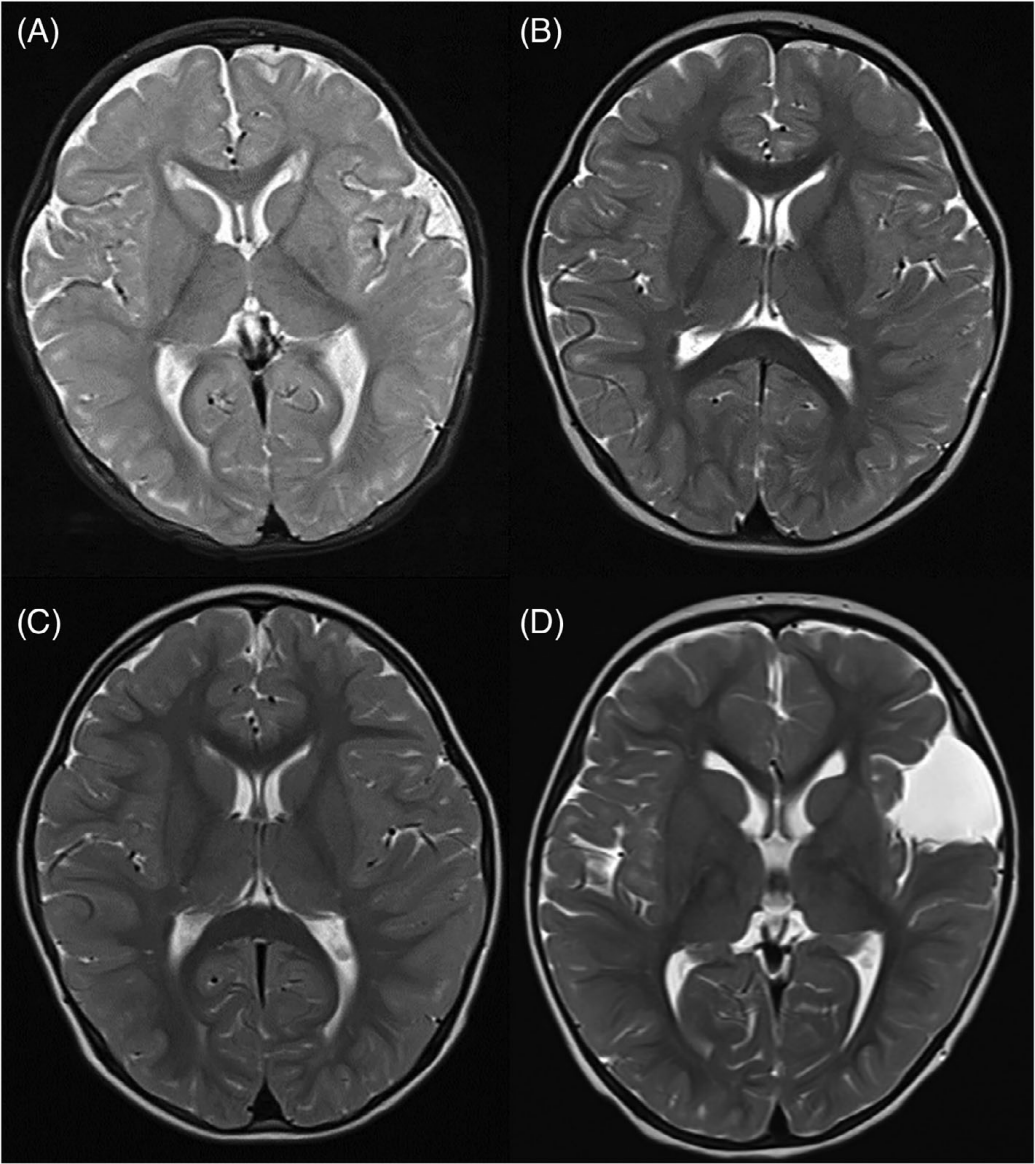
MRI brain (T2‐weighted images) in P1; (A) at 12 months of age showing delayed myelination in cerebral hemispheres, (B) at 3 years of age showing some ‘catch‐up’ of myelination, with incomplete myelination in several gyri, and (C) at 4.75 years of age, 18 months after commencing triheptanoin, showing almost age‐appropriate myelination. (D) MRI brain (T2‐weighted images) in P2 at 3.2 years of age, showing incompletely myelinated cerebral gyri, mild global volume loss and a left frontotemporal arachnoid cyst.

Growth and developmental progress in infancy were normal, however mild persistent hyperlactataemia was observed (range 4.1–5.9 mmol/L, normal < 2.2 mmol/L). Blood lactate/pyruvate ratio was mildly elevated (range 24–29, normal < 25). Exacerbation of hyperlactataemia without acidosis occurred during viral respiratory infections at 6 and 8 months of age, requiring hospitalisation for intravenous glucose. MRI brain at 12 months of age showed delayed cerebral myelination (Figure 1A).

A further viral respiratory infection at 13 months of age resulted in profound lactic acidosis requiring haemofiltration. Following discharge, an oral nutritional supplement (1 kcal/mL) was commenced to ensure adequate energy during periods of poor appetite. An emergency regimen (ER) comprising frequent glucose polymer (15% carbohydrate solution) feeds was advised, to provide energy during intercurrent illnesses.

Compound heterozygous *PC* variants NM_000920.3:c.1358G>A, p.(Arg453Gln); c.865_870del, p.(Thr289_Arg290del) were detected by trio whole exome sequencing at 18 months of age, and his phenotype was considered to be consistent with PCD type C. Tricitrate (5 mmol/kg/day of alkali) was commenced, biotin was increased to 20 mg/day, and thiamine was continued.

At 23 months of age he required fluid resuscitation and intravenous glucose due to symptomatic lactic acidosis during a febrile illness. Riboflavin 50 mg/day was commenced at 2 years of age.

At 2.5 years of age a history of recurrent exertional dyspnoea was reported, and he was found to have a post‐exertional rise in blood lactate (Table 2). Speech development was markedly delayed and he had a broad‐based, toe‐walking gait; however, neurological examination was otherwise normal. Repeat MRI brain at 3 years of age showed evidence of interval progression of myelination (Figure 1B).

**TABLE 2:** Venous blood lactate at baseline and during treatment with triheptanoin (+time since initiation), measured at rest and immediately following exercise (blood lactate reference range <2.2 mmol/L).

Venous blood lactate	Baseline (+0 months)	Triheptanoin 30 g/day (+3 months)	Triheptanoin 35 g/day (+13 months)
Pre‐exercise	2.7 mmol/L	3.1 mmol/L	3.5 mmol/L
Post‐exercise	6.2 mmol/L	5.1 mmol/L	2.6 mmol/L

### 2.2 Patient 1; triheptanoin treatment

A therapeutic trial of triheptanoin was commenced at 3.25 years of age. Initially, a diet diary was completed to assess daily energy and long‐chain fat intake. The liquid medication (triheptanoin 100% w/w) was given in four divided doses with meals, diluted with juice or skimmed milk to improve palatability. It was commenced during an elective 3‐day admission. The dose was then gradually up‐titrated, due to transient diarrhoea. The target dose was 35 mL/day (1.85 g/kg/day), providing 25% of daily energy intake, based on dosages used in LC‐FAOD in the literature and in our centre's experience.^15^, ^16^, ^17^ Dietary long‐chain triglycerides were gradually restricted to 10% of daily energy intake. Thus, 35% of total daily energy was from fat, in accordance with recommendations for the general population. Fat‐soluble vitamin supplements and walnut oil for essential fatty‐acids were prescribed.

During up‐titration of the triheptanoin dose, one further exacerbation of severe lactic acidosis requiring hospitalisation occurred, while receiving 30 mL/day. This followed a two‐week history of viral respiratory symptoms, recurrent vomiting and reduced intake, during which time the dose was reduced to 10 mL/day. Intravenous glucose was required for 4 days, followed by a quick recovery. Triheptanoin was suspended for a total of 3 weeks, due to potential gastrointestinal intolerance, and was then restarted at a lower dose (5 mL/day) with gradual escalation over the next 3 months.

Once the target dose of 35 mL/day was reached at 3.75 years of age, no further episodes of metabolic decompensation occurred during 2 years of follow‐up. Subsequent viral infections were well tolerated, and were manageable at home using the ER. At 4 years of age, he presented to hospital with a history of poor intake and vomiting over several days; however, blood gas and lactate were normal and he was discharged home.

Post‐exertional hyperlactataemia resolved once the target dose of triheptanoin was reached (Table 2). This was associated with an improvement in exercise tolerance, allowing increased participation in active play without episodes of dyspnoea.

Repeat MRI brain 18 months after initiation of triheptanoin showed improved myelination (Figure 1C), and continued developmental progress was observed during treatment. Standardised developmental assessments were administered at 4.9 years of age, and a language disorder and speech disorder impacted by poor oro‐motor skills were diagnosed. Visual working memory and non‐verbal reasoning were in the low‐average range.

### 2.3 Patient 2

Patient 2 (P2) was the first child to non‐consanguineous parents, and was born by caesarean section at 36/40. She was admitted to the neonatal unit for 2 days due to respiratory distress, requiring non‐invasive respiratory support and antibiotics. However, there was no significant neonatal hypoglycaemia or acidosis. Gross motor milestones were delayed, with standing attained at 20 months and walking at 24 months. However, growth was normal and she remained generally well in the first 2 years.

At 2 years of age she presented with laboured breathing, poor perfusion and reduced alertness, after 24 h of vomiting with minimal intake. She had profound metabolic acidosis with hyperlactataemia and ketonuria, but no hypoglycaemia (Table 1). Cranial computed tomography scan showed a left frontotemporal arachnoid cyst, which was not considered relevant to her acute presentation. Following fluid resuscitation with normal saline, sodium bicarbonate and glucose, blood ketones remained persistently elevated 24 h after presentation, at 2.6 mmol/L. She then made a full recovery and was discharged home 4 days later. An ER (20% carbohydrate solution from glucose polymer) was prescribed prior to discharge.

At 2.5 years of age, she developed another episode of severe keto‐lactic acidosis with hypoglycaemia, after waking with repetitive vomiting and lethargy. She was treated with intravenous glucose, saline, and sodium bicarbonate, and was discharged the following day.

Compound heterozygous *PC* variants NM_001040716.1:c.2767C>G, p.(Gln923Glu); c.2800del p.(Gln934Argfs*35) were detected by trio whole genome sequencing at 3 years of age. Pyruvate carboxylase activity was then measured in cultured skin fibroblasts, and was 7.5 nmol/h/mg protein, equivalent to 8% of the mean of four unaffected controls measured simultaneously (94 ± 56.9 nmol/h/mg protein).

At 3 years of age, gross motor development remained delayed with no running or jumping, and concerns regarding poor balance and frequent falls. She was able to speak in four‐word sentences, and there were no concerns regarding other developmental domains. There was no history of exertional dyspnoea, and blood gases following exercise did not demonstrate a lactate rise. MRI brain at 3.2 years of age showed mild global volume loss, and stable appearance of the known left frontal cystic lesion (Figure 1D).

Following diagnosis, biotin 20 mg/day and tricitrate (5.7 mmol/kg/day of alkali) were initiated. Triheptanoin was commenced at 3.5 years of age, and was well tolerated with only one instance of loose stools. The target dose (35 mL/day, 25% of daily energy) was reached after 5 weeks. Long‐chain triglycerides were restricted and micro‐nutrient supplements were prescribed as for P1. No further acute decompensations occurred since treatment was commenced.

## 3 DISCUSSION

Compared to PCD types A/B, type C is extremely rare, with 13 patients reported including the two presented here. It is typically associated with late‐onset milder manifestations, however these patients demonstrate the variable age of onset. Neonatal lactic acidosis has been reported previously in PCD type C^7^; however, P1 developed severe lactic acidosis and hypoglycaemia on the first day of life, which is the earliest reported presentation. In both patients, recognition of this rare clinical entity was challenging, and other causes of recurrent lactic acidosis and ketoacidosis were initially considered. In PCD types A/B, the diagnosis may be suspected on the basis of characteristic biochemical findings which may include increased lactate/pyruvate and acetoacetate/β‐hydroxybutyrate ratios, hyperammonaemia, elevated plasma alanine, citrulline and lysine and low plasma glutamine and aspartate.^1^, ^2^ These findings are not consistently observed in PCD type C^1^, ^2^; however, mildly elevated plasma alanine with low aspartate and glutamine were present in both P1 and P2, and may be useful clues to the diagnosis.

Increased availability of next generation sequencing is likely to improve detection of this disorder, as demonstrated in P1 and P2. Both were compound heterozygous for previously unreported *PC* variants, that were also absent in the gnomAD database of healthy individuals. The c.865_870del variant in P1 is predicted to generate an in‐frame deletion of two amino acid residues resulting in a truncated protein, and the c.1358G>A missense variant affects a highly conserved residue in the critical biotin carboxylase domain, and is predicted to be pathogenic by in silico algorithms (SIFT, PolyPhen). The c.2800del frameshift variant in P2 is predicted to cause nonsense‐mediated decay, and the missense variant c.2767C>G is predicted to be pathogenic in silico. The *PC* gene has a low rate of benign missense variation, with a significantly raised ExAC constraint score (*z* = 4.05), and this was used as further evidence supporting the pathogenicity of these variants, in addition to confirmation of bi‐allelic inheritance, and the specific phenotype. PC enzymology was performed to support the diagnosis in P2, and demonstrated deficient activity, 8% of normal controls.

However, residual PC activity correlates poorly with clinical severity, and thus PCD is classified on the basis of clinical presentation.^1^, ^10^, ^19^ PCD types A and B are typically associated with Leigh disease or fatal neonatal encephalopathy, whereas type C may be associated with a relatively preserved neuro‐developmental outcome.^1^, ^3^, ^5^ Neurological manifestations are well‐reported however, including variable developmental delay, episodic hemiplegia, acute flaccid paralysis, hypomyelination, and leukodystrophy.^6^, ^7^, ^8^, ^9^ P1 had a severe speech impairment, and both P1 and P2 had gross motor delay, gait abnormalities and cerebral hypomyelination, emphasising that PCD type C should not be considered benign.

It has been suggested that abnormal myelination may contribute to the neurological phenotype, as a result of impaired fatty acid synthesis from citrate.^1^, ^7^ Triheptanoin has been shown to generate anaplerotic substrates in CSF that have potential to improve cerebral synthesis of fatty acids and myelin.^14^ It was encouraging in P1 that myelination improved on MRI after 18 months of triheptanoin treatment, associated with continued developmental progress.

Exercise intolerance due to exertional lactic acidosis was seen in P1 but not P2, and is not a commonly reported feature in PCD. It is seen in respiratory chain defects, and in PCD it presumably reflects variable TCA cycle impairment in muscle. A significant symptomatic improvement was demonstrated during triheptanoin treatment, and was corroborated by a dose‐dependent reduction in post‐exertional hyperlactataemia. This may be due to provision of anaplerotic TCA substrates to muscle.

Finally, a marked reduction in the frequency of metabolic decompensations requiring hospitalisation was observed once the full triheptanoin dose was reached. This was a clinically meaningful outcome, however it could be coincidental and thus should be replicated in further patients. Biotin and tricitrate were given throughout the trial of triheptanoin; however, these were started long before the observed reductions in metabolic crises and exertional hyperlactataemia. The efficacy of triheptanoin in prevention of metabolic crises may be impacted by reduced gastro‐intestinal tolerability during infections, and maintenance of adequate energy intake during illness remains an essential component of treatment.

## 4 CONCLUSION

Triheptanoin was well‐tolerated and appeared efficacious during 2 years' follow‐up in a patient with PCD type C, and has been safely initiated in our second patient. These encouraging findings merit further investigation.

## FUNDING INFORMATION

Julien Baruteau is supported by funding from the United Kingdom Medical Research Council Clinician Scientist Fellowship (MR/T008024/1) and NIHR Great Ormond Street Hospital Biomedical Research Centre. The views expressed are those of the author(s) and not necessarily those of the NHS, the NIHR or the Department of Health.

## CONFLICT OF INTEREST STATEMENT

The authors have no conflict of interest to declare.

## ETHICS STATEMENT

Written consent for publication was obtained from patients' parents.

## Data Availability

The authors do not have authorisation from the families to share personal data. Sharing anonymised data can be discussed upon request.

## References

[1] Marin‐Valencia I , Roe CR , Pascual JM . Pyruvate carboxylase deficiency: mechanisms, mimics and anaplerosis. Mol Genet Metab. 2010;101(1):9‐17. 20598931 10.1016/j.ymgme.2010.05.004

[2] Wang D , De Vivo D . Pyruvate carboxylase deficiency. In: Adam MP et al., eds. GeneReviews® [Internet]. University of Washington, Seattle; 2009. 20301764

[3] Van Coster RN , Fernhoff PM , De Vivo DC . Pyruvate carboxylase deficiency: a benign variant with normal development. Pediatr Res. 1991;30(1):1‐4. 1909777 10.1203/00006450-199107000-00001

[4] Higgins JJ , Glasgow AM , Lusk M , Kerr DS . MRI, clinical, and biochemical features of partial pyruvate carboxylase deficiency. J Child Neurol. 1994;9(4):436‐439. 7822739 10.1177/088307389400900421

[5] Hamilton J , Rae MD , Logan RW , Robinson PH . A case of benign pyruvate carboxylase deficiency with normal development. J Inherit Metab Dis. 1997;20(3):401‐403. 9266366 10.1023/a:1005350600278

[6] Arnold GL , Griebel ML , Porterfield M , Brewster M . Pyruvate carboxylase deficiency. Report of a case and additional evidence for the “mild” phenotype. Clin Pediatr. 2001;40(9):519‐521. 10.1177/000992280104000909 11583052

[7] Schiff M , Levrat V , Acquaviva C , et al. A case of pyruvate carboxylase deficiency with atypical clinical and neuroradiological presentation. Mol Genet Metab. 2006;87(2):175‐177. 16325442 10.1016/j.ymgme.2005.10.007

[8] Wang D , Yang H , De Braganca KC , et al. The molecular basis of pyruvate carboxylase deficiency: mosaicism correlates with prolonged survival. Mol Genet Metab. 2008;95(1–2):31‐38. 18676167 10.1016/j.ymgme.2008.06.006 PMC2572257

[9] Almomen M , Sinclair G , Stockler‐Ipsiroglu SG , Horvath GA . Pyruvate carboxylase Deficiency type C: a rare cause of acute transient flaccid paralysis with ketoacidosis. Neuropediatrics. 2018;49(6):369‐372. 30045381 10.1055/s-0038-1667171

[10] Coci EG , Gapsys V , Shur N , et al. Pyruvate carboxylase deficiency type a and type C: characterization of five novel pathogenic variants in *PC* and analysis of the genotype–phenotype correlation. Hum Mutat. 2019;40:816‐827. 30870574 10.1002/humu.23742

[11] Doğulu N , Öncül Ü , Köse E , Aycan Z , Eminoğlu FT . Pyruvate carboxylase deficiency type C as a differential diagnosis of diabetic ketoacidosis. J Pediatr Endocrinol Metab. 2021;34(7):947‐950. 33860652 10.1515/jpem-2020-0646

[12] Higgins JJ , Ide SE , Oghalai JS , Polymeropoulos MH . Lack of mutations in the biotin‐binding region of the pyruvate carboxylase (PC) gene in a family with partial PC deficiency. Clin Biochem. 1997;30(1):79‐81. 9056115 10.1016/s0009-9120(96)00125-7

[13] Ahmad A , Kahler SG , Kishnani PS , et al. Treatment of pyruvate carboxylase deficiency with high doses of citrate and aspartate. Am J Med Genet. 1999;87(4):331‐338. 10588840 10.1002/(sici)1096-8628(19991203)87:4<331::aid-ajmg10>3.0.co;2-k

[14] Mochel F , DeLonlay P , Touati G , et al. Pyruvate carboxylase deficiency: clinical and biochemical response to anaplerotic diet therapy. Mol Genet Metab. 2005;84(4):305‐312. 15781190 10.1016/j.ymgme.2004.09.007

[15] Roe CR , Brunengraber H . Anaplerotic treatment of long‐chain fat oxidation disorders with triheptanoin: review of 15 years experience. Mol Genet Metab. 2015;116(4):260‐268. 26547562 10.1016/j.ymgme.2015.10.005 PMC4712637

[16] Zöggeler T , Stock K , Jörg‐Streller M , et al. Long‐term experience with triheptanoin in 12 Austrian patients with long‐chain fatty acid oxidation disorders. Orphanet J Rare Dis. 2021;16(1):28. 33446227 10.1186/s13023-020-01635-x PMC7807521

[17] Vockley J , Burton BK , Berry G , et al. Triheptanoin for the treatment of long‐chain fatty acid oxidation disorders: final results of an open‐label, long‐term extension study. J Inherit Metab Dis. 2023;46(5):943‐955. 37276053 10.1002/jimd.12640

[18] Breen C , White FJ , Scott CA , et al. Unsuccessful treatment of severe pyruvate carboxylase deficiency with triheptanoin. Eur J Pediatr. 2014;173(3):361‐366. 24114256 10.1007/s00431-013-2166-5

[19] Stern HJ , Nayar R , Depalma L , Rifai N . Prolonged survival in pyruvate carboxylase deficiency: lack of correlation with enzyme activity in cultured fibroblasts. Clin Biochem. 1995 Feb;28(1):85‐89. 7720232 10.1016/0009-9120(94)00059-5

